# Unit cost of healthcare services at 200-bed public hospitals in Myanmar: what plays an important role of hospital budgeting?

**DOI:** 10.1186/s12913-017-2619-z

**Published:** 2017-09-19

**Authors:** Thet Mon Than, Yu Mon Saw, Moe Khaing, Ei Mon Win, Su Myat Cho, Tetsuyoshi Kariya, Eiko Yamamoto, Nobuyuki Hamajima

**Affiliations:** 1 Department of Healthcare Administration, Nagoya University Graduate School of Medicine, 65 Tsurumai-cho, Showa-ku, Nagoya, 466-8550 Japan; 2 Medical Care Division, Department of Medical Services, Ministry of Health and Sports, Nay Pyi Taw, Myanmar; 3 Nagoya University Asian Satellite Campuses Institute, Nagoya, Japan; 4 Occupational and Environmental Health Division, Department of Public Health, Ministry of Health and Sports, Nay Pyi Taw, Myanmar

**Keywords:** Unit cost, Healthcare services, Public hospitals, Government health expenditure, Myanmar

## Abstract

**Background:**

Cost information is important for efficient allocation of healthcare expenditure, estimating future budget allocation, and setting user fees to start new financing systems. Myanmar is in political transition, and trying to achieve universal health coverage by 2030. This study assessed the unit cost of healthcare services at two public hospitals in the country from the provider perspective. The study also analyzed the cost structure of the hospitals to allocate and manage the budgets appropriately.

**Methods:**

A hospital-based cross-sectional study was conducted at 200-bed Magway Teaching Hospital (MTH) and Pyinmanar General Hospital (PMN GH), in Myanmar, for the financial year 2015–2016. The step-down costing method was applied to calculate unit cost per inpatient day and per outpatient visit. The costs were calculated by using Microsoft Excel 2010.

**Results:**

The unit costs per inpatient day varied largely from unit to unit in both hospitals. At PMN GH, unit cost per inpatient day was 28,374 Kyats (27.60 USD) for pediatric unit and 1,961,806 Kyats (1908.37 USD) for ear, nose, and throat unit. At MTH, the unit costs per inpatient day were 19,704 Kyats (19.17 USD) for medicine unit and 168,835 Kyats (164.24 USD) for eye unit. The unit cost of outpatient visit was 14,882 Kyats (14.48 USD) at PMN GH, while 23,059 Kyats (22.43 USD) at MTH. Regarding cost structure, medicines and medical supplies was the largest component at MTH, and the equipment was the largest component at PMN GH. The surgery unit of MTH and the eye unit of PMN GH consumed most of the total cost of the hospitals.

**Conclusion:**

The unit costs were influenced by the utilization of hospital services by the patients, the efficiency of available resources, type of medical services provided, and medical practice of the physicians. The cost structures variation was also found between MTH and PMN GH. The findings provided the basic information regarding the healthcare cost of public hospitals which can apply the efficient utilization of the available resources.

**Electronic supplementary material:**

The online version of this article (10.1186/s12913-017-2619-z) contains supplementary material, which is available to authorized users.

## Background

Cost information helps managers and decision makers to improve the medical services quality and to make the cost projections [1]. Hospital cost supports to strengthen the efficient allocation of resources and hospital performance [1]. It also provides basic information to set user fees with the acceptable level of quality and price for the community. In addition, it could be useful to implement a prepayment mechanism as a way towards universal health coverage (UHC), for which many countries are trying to develop owned financing systems [2].

Hospitals are essential and important to the healthcare system. They provide both health service delivery and training of health professionals. Hospitals are also the largest financing source as they utilize money for professional human resources and medical equipment. Furthermore, they provide primary healthcare and receive referral cases for advanced cases. In recent years, studies of cost analysis have been increasing because the understanding on cost structures of hospitals is essential to improve the efficiency and quality of healthcare services [3]. World Health Organization has a project of providing cost information in member states for policy makers to guide efficient allocation of health expenditure [4].

South Africa and India—where utilization of hospitals is increased and health system strengthening is needed— conducted the unit cost studies. These studies reported that the cost for human resource represented the largest among all of the cost components [5, 6]. In Southern Ghana, a study estimating unit costs and cost components was also conducted at three hospitals of different types for year 2002 and 2003 in order to understand health system efficiencies at the provider level. This study found that the direct cost of pharmacy department, including human resource working in that department, was the largest cost component [7]. To develop a financing strategy for policy implication of efficient utilization of scarce resources, a study was conducted at hospitals and primary health centers in Palestine for the year 2008. It found that salaries accounted for the majority portion of total cost, followed by drugs and consumables cost and other expenditure [8].

In Myanmar, the reform process of health sector was introduced in March 2011 [9]. Since then, the total government health expenditure has been increasing—7688 million Kyats (5.66 million USD), which was 0.20% of GDP, in financial year 2000–2001 to 652,745 million Kyats (480.31million USD), which was 0.99% of GDP, in financial year 2014–2015 [10]. Myanmar allocated 3.65% of its total budget on health in financial year 2016–2017 (850 million USD), which was a nine-fold increase in absolute amount in financial year 2010–2011 (94 million USD) [11]. The budget was mainly used to finance delivery of healthcare, expanding the service coverage with a focus on free medical care in hospital settings [11]. The efficient allocation of the increased government health expenditure is important for a developing country with limited resources. In Myanmar, most of the government health expenditure (69.80% in financial year 2011–2012) invested in public hospitals [12].

Cost studies have been few in Myanmar to examine the effectiveness of health expenditure and the efficiency of hospitals. To evaluate the efficiency, cost information is required from different financial and cost aspects of the financial system, including budgeting and accounting [13]. The government health expenditure is low in low-income countries, and therefore, cost of health services is mainly dependent on private household expenditure. In Myanmar, out-of-pocket (OOP) payment of individual households has been a major source of healthcare financing since 1990s [14]. As high expenditure of health services can lead to financial catastrophe, sick people may later face financial hardship. This could potentially worsen their health. Higher cost also prevents individuals from seeking healthcare, and thus, this lead to them being unhealthy. It is important to know the cost of health services and the efficiency of available resources for providing these services at an accessible cost. Thus, the Myanmar government has been working to reduce the individual household’s financial burden of healthcare services [15].

In Myanmar, curative services are provided mostly at public hospitals, including general, specialty, teaching, region/state level, district level, township level, and station hospitals. Station hospitals are the smallest medical units with general medical, surgical, and obstetrics services, besides some diagnosis facilities. Specialty services are accessible, starting from district level hospitals to some township hospitals in special areas. The central or national level hospitals provide tertiary care with more advanced healthcare services, and region/state level hospitals provide secondary care [16]. The number of hospital beds varies from 16 beds to 2000 beds depending on the level of the hospital.

The total number of public hospitals is 1123 in Myanmar, wherein more than 1000 hospitals are station to district level hospitals. There are 27 hospitals with 200 beds that constitute mostly region/state level hospitals and 9 teaching hospitals [17]. Private sector coverage is limited because the private sector mostly provides ambulatory care and some institutional care are only available for major cities. There are only 214 private hospitals, and they have different structures and functions. Hospital budget allocation is decided by the central government based on the needs of each hospital. It also relies on the union government policy [18].

In Myanmar, although government health expenditure has seen a moderate increase, the efficiency and effectiveness of this increased budget is still controversial. Furthermore, identifying the basic essential package of health services, further establishing the health insurance system, and decreasing the cost of health services for better accessibility also requires an urgent investigation into health services expenditures. To the best of our knowledge, our study, which assessed the unit cost of health services at 200-bed public hospitals, has so far never been conducted in Myanmar. Therefore, we aimed to calculate the unit cost of healthcare services at two 200-bed public hospitals in two different regions to provide basic information on the appropriate allocation and management of budgets. The hospitals under study were a regional level hospital, Pyinmanar General Hospital (PMN GH), and a national level hospital, Magway Teaching Hospital (MTH). The cost structure and cost consuming pattern of the healthcare services were also examined in this study.

## Methods

### Study setting

PMN GH is a secondary level hospital located in Nay Pyi Taw Union Territory. It was established in 1933 as Bombay Barmah hospital, and then, upgraded to a 200-bed regional level hospital in 2007. MTH is located in Magway region, which is in the central region of Myanmar. MTH is a tertiary level hospital that was established in 2003 as a teaching hospital for the University of Medicine, Magway. These two hospitals were selected by following criteria: 1) accessibility of hospital data, including detailed hospital expenditure and service data; 2) willingness to cooperate; and 3) reasonable hospital utilization rate. Specialty hospitals were excluded from this study due to their special procedures, treatments, and different cost structures. The private hospitals were also excluded from this study because of their different structure and functions.

### Data collection

Annual data of the financial year 2015–2016 (1 April, 2015 to 31 March, 2016) were collected in September 2016 by researchers using prepared pro forma. The data collection process included three parts: 1) reviewing the hospital administrative records and logistics information, 2) identifying potential interviewees, and 3) conducting face-to-face interviews with responsible staff for data validation. The collected data included basic characteristics and performance of hospital, capital assets, salary and allowances of staff, medicines and medical supplies, numbers of equipment and its cost, and other recurrent costs.

### Costing method

The step-down method, also known as the top-down method, was applied for the study. The step-down method calculates the unit cost of healthcare services by allocation of the total hospital cost that includes seven steps for computing unit cost. The seven steps were 1) defining the final products, 2) defining the cost centers, 3) identifying the cost for each input, 4) assigning the cost of each input to cost centers, 5) allocation of all costs to final cost centers, 6) computing the unit cost, and 7) reporting the results [19].

### Defining the final products

First, the final products of the study were assigned as unit cost of inpatient (number of admission cases and inpatient days) and outpatient (outpatient visits) services, and unit cost of operation (number of operated patients) according to the health services provided by each hospital, with the purpose of measuring the unit cost of each department.

### Defining cost centers

Cost centers with three levels were developed. These were 1) overhead cost center, 2) intermediate cost centers, and 3) final cost centers, each based on their function from an administrative standpoint. Overhead cost center included the administrative department. The intermediate cost centers were pharmacy, laboratory, radiology, intensive care unit (ICU), operation theatre, physical medicine, and rehabilitation department. The final cost centers were all inpatient and outpatient departments (OPD). The departments comprised of 1) medicine; 2) surgery; 3) obstetrics and gynecology (OG); 4) pediatrics; 5) orthopedics; 6) eye, 7) ear, nose, and throat (ENT); 8) general OPD; 9) dental unit at both hospitals, and 10) oncology, and 11) specialist OPD only at MTH. In addition, MTH has a dispensary for outpatients of general OPD and specialist OPDs. Therefore, the separate unit cost for general and specialist OPD cases, each, were calculated for MTH. Moreover, MTH had an oncology department, but it was not available at PMN GH. Similarly, PMN GH had an ENT unit, which was not available at MTH.

### Identifying the cost for each input

In the third step, the cost of the inputs was categorized into capital and recurrent costs. The government provides the budget by line items, which were identified in the study. Interviews with relevant staff and record review were also done to identify items other than these line items.

Capital assets have an economically useful life, which is defined as being used longer than one year [20]. The capital assets of the hospitals included buildings, major equipment, vehicles, and furniture. Cost analysis of capital assets used annual capital costs instead of original costs because the assets are depleted on a daily basis with the hospital’s regular routine. This depleted value is known as depreciation, which was considered in calculating the annual capital cost [19].

The annual capital cost was obtained from the division of the replacement cost of the assets for the relevant year by the annualization factor [19]. Replacement cost for the relevant year was calculated based on the interest rate on the cost of the assets. We applied a 3.00% interest rate for all capital assets based on the data of Central Bank of Myanmar. The annualization factor was defined based on the discount rate and the total life of the asset [19]. In this study, total life was set as 50 years for buildings, 16 years for generators, 15 years for furniture (except office wooden chairs), 10 years for air-conditioners, five years for diagnostic imaging machines and rehabilitative therapy machines, eight years for other medical equipment, eight years for vehicles, eight years for office wooden chairs, and five years for office equipment, including computer and accessories, based on the official orders of Myanmar government. The discount rate was taken as 3.00% for all capital assets, and it was calculated by using a formula based on nominal interest rate and annual inflation rate [19]. Among capital assets, land was not considered as a depreciable item, because it can maintain its value, although it still has opportunity costs [21]. Therefore, land cost was not included in the study; it is also beyond the control of the hospital administrators [6].

Regarding the recurrent costs, staff salaries and allowances, medicines and medical supplies, consumables, recurrent equipment items, and other costs for daily activities were included. Staff salaries were calculated using the average salary for each rank of staff. For allowances, civil servant general allowance, uniform fees, travel cost, and housing allowance were included. Quantity for medicines and medical supplies were collected from main stock books of the pharmacy department, and main and sub-stock books of each department. The costs of these medicines and medical supplies were calculated according to the price list of each source of supply. The equipment list was obtained mostly from the non-expenditure register of hospitals and list from each ward. The costs of donated items were included in the study, and replacement cost was used for calculating the cost of these items. Other variable costs, such as transport and labour cost, stationery, fuel use, telephone bill, printing, taxes, electricity, utilities, food supplies, maintenance, and others, were obtained from the record of the administrative department and each unit.

### Assigning inputs to cost centers

After identifying the full cost of each resource item, each cost was assigned to the respective cost centers according to a predefined set of rules. Some costs were assigned directly into each cost center [6]. The rules for assigning inputs to each cost center are described in Additional file 1: Table S1.

### Allocation of all costs to final cost centers

The costs of overhead and intermediate cost centers were allocated to final cost centers by allocation criteria, which were appropriate for study hospitals [22], in a stepwise process. The ICUs were under the supervision of anesthetists, and its functions were together done by the operation theatre. Therefore, the costs of ICUs were allocated to the operation theatre. At PMN GH, although the ICU was available, it was not in function. At MTH, ICU had an output of only two inpatient days within the financial year 2015–2016, and most patients were temporarily monitored in the ICU after their operations. Additional file 2: Table S2 describes the rules for allocation.

### Computing unit cost

In the final step, for each final cost center, the total and unit cost of final products were calculated according to hospital statistics—inpatient days, cases (number of admission and operation) and out-patient visits, and analyzed cost consuming pattern and cost structures. The unit cost per operation case, unit cost per radiology case, and unit cost per case at the physical medicine and rehabilitation department were calculated using their total cost before allocation to final cost centers. The costs in US dollar were shown for reference in the currency rate of 2 April, 2015 (1 USD = 1028 Kyats) [23]. The data were processed and analyzed using Excel spreadsheets (Microsoft Excel 2010).

## Results

### Basic characteristics of the study hospitals

The bed occupancy rate of PMN GH was 83.35% for sanctioned beds and 61.74% for available beds, while it was 93.14% for sanctioned beds and 76.03% for available beds for MTH. The average total number of staff per month during the period under study was 232 at PMN GH, and 288 at MTH, which included teaching staff as well. The inpatient days and the number of operation cases were higher at MTH than PMN GH. However, the total number of admission cases and outpatient visits were higher at PMN GH (Table 1).

**Table 1:** Basic characteristics of Pyinmanar General Hospital (PMN GH) and Magway Teaching Hospital (MTH) in 2015–2016

Basic characteristic	PMN GH	MTH
Sanctioned beds	200	200
Available beds	270	245
Total number of staff^a^	232	288
Bed occupancy rate for sanctioned beds	83.35%	93.14%
Bed occupancy rate for available beds	61.74%	76.03%
Inpatient days^b^	60,844	67,991
Outpatient visits	59,882	26,683
Admissions	15,504	11,369
Operations	3118	3743
Major operations	1319	2761
Minor operations	1799	982
Laboratory tests	21,740	71,325
Blood transfusions	2260	3308
Patients examined by a X-ray test	10,502	6944
X-ray film used	13,996	9388
Patients examined by an ultrasound test	5671	0

^a^At PMN GH; Consultant doctors 6.8%; Medical officers 13.6%; Nurses 37%; Medical technician 8.9%; others - 33.6%; At MTH; Consultant doctors 5.1%; Medical officers 13.4%; Nurses 44.5%; Medical technician 5.7%; others 20.9%; Teaching staff (Professor, Associate Professor, Lecturer, Assistant Lecture) 2.7%; Post graduate students 7.8%

^b^A day of inpatient care provided to a patient in a hospital which begins at midnight and ends 24 h later at the next midnight [33]

### Unit cost of each cost center of the study hospitals

Tables 2 and 3 present the unit costs of the healthcare services, which included two types of unit—1) per inpatient day, and 2) per case according to the major inpatient wards, outpatient departments, and operation theatre. The unit costs per inpatient day varied from 28,374 Kyats (27.60 USD) for the pediatric unit to 1,961,806 Kyats (1908.37 USD) for the ENT unit at PMN GH. For MTH, the unit costs per inpatient day varied from 19,704 Kyats (19.17 USD) for the medicine unit to 168,835 Kyats (164.24 USD) for the eye unit.

**Table 2:** Unit cost of inpatient units at Pyinmanar General Hospital (PMN GH) and Magway Teaching Hospital (MTH) in 2015–2016

Cost center	Unit	PMN GH			MTH		
		Output	Unit cost		Output	Unit cost	
			Kyats	USD		Kyats	USD
Medicine	Inpatient day	11,091	35,425	34.5	17,195	19,704	19.2
	Case	3554	110,552	107.5	2702	125,392	121.9
Surgery	Inpatient day	10,214	35,676	34.7	18,925	39,483	38.4
	Case	2719	134,017	130.4	2967	251,842	244.9
Obstetrics and gynecology	Inpatient day	12,265	34,602	33.7	11,605	31,668	30.8
	Case	3249	130,621	127.1	2051	179,182	174.3
Pediatrics	Inpatient day	13,454	28,374	27.6	7915	21,587	20.9
	Case	3325	114,759	111.6	1421	120,239	116.9
Orthopedics	Inpatient day	11,828	35,291	34.3	11,372	36,733	35.7
	Case	1695	246,265	239.6	1151	362,925	353.0
Eye	Inpatient day	1911	278,259	270.7	977	168,835	164.2
	Case	945	562,701	547.4	502	328,589	319.6
ENT^a^	Inpatient day	81	1,961,806	1908.4			
	Case	17	9,347,429	9092.8			
Oncology^b^	Case				575	260,869	253.8

1 USD = 1028 kyats (on 2nd April, 2015)

^a^Ear, nose and throat

^b^During the study period, oncology patients were admitted to inpatient unit which referred them to oncology unit. So, the unit cost per inpatient day for oncology unit was not calculated

**Table 3:** Unit cost of outpatient departments and intermediate cost centres at Pyinmanar General Hospital (PMN GH) and Magway Teaching Hospital (MTH) in 2015–2016

Cost center	Unit	PMN GH			MTH		
		Number of services	Unit cost		Number of services	Unit cost	
			Kyats	USD		Kyats	USD
Dental	Case	1352	22,630	22.0	2638	33,451	35.5
OPD^a^	Visit	59,882	14,882	14.5	26,683	23,059	22.4
General OPD^a^	Visit				5734	28,691	27.9
Specialist OPD^a^	Visit				23,122	21,287	20.7
PM & R^b^	Case	121	41,345	40.2	923	26,064	25.4
Operation theatre							
Major operation	Case	1319	277,519	269.9	2761	272,639	265.2
Minor operation	Case	1799	55,504	53.9	982	54,528	53.04
Radiology							
X-ray	Case	10,502	14,325	13.9	6944	147,609	143.6
Ultrasound	Case	5671	28,650	27.9			
Laboratory							
Laboratory test	Test	21,740	3988	3.9	71,325	673	0.7
Blood transfusion	Unit	2260	25,577	24.9	3308	7814	7.6

1 USD = 1028 Kyats (on 2nd April, 2015)

^a^Outpatient department

^b^Physical medicine and rehabilitation

As outpatient units, the unit cost of outpatient case was 14,882 Kyats (14.48 USD) at PMN GH, and 23,053 Kyats (22.43 USD) at MTH. The unit costs for operation theatre were 277,519 Kyats (269.96 USD) per major operation case and 55,504 Kyats (53.99 USD) per minor operation case at PMN GH, and 272,639 Kyats (265.21 USD) per major operation case and 54,528 Kyats (53.04 USD) per minor operation case at MTH. The unit costs per X-ray patient was 14,325 Kyats (13.93 USD) at PMN GH and 147,609 Kyats (143.59 USD) at MTH. At PMN GH, the unit cost per laboratory test was 3988 Kyats (3.88 USD), while it was only 673 Kyats (0.66 USD) at MTH.

### Cost structures of study hospitals

**Fig. 1:**
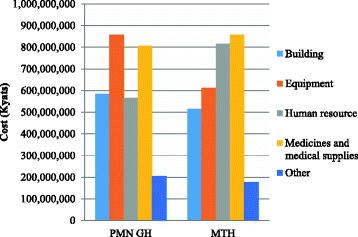
Cost components of Pyinmanar General Hospital (PMN GH) and Magway Teaching Hospital (MTH) in 2015–2016. The cost components were divided into buildings, human resource, medicines and medical supplies, equipment, and others. The medicines and medical supplies represented the largest cost component at MTH, and the second largest at PMN GH. The equipment cost was the largest component at MTH, while it takes the third position at PMN GH. The human resource represented the second largest cost component at MTH, and the fourth largest at PMN GH

In terms of the cost structures of the hospitals, the total cost was 3,019,436,643 Kyats for PMN GH and 2,978,343,682 Kyats for MTH. The cost was partly covered by donation (1.63% and 0.41%, respectively), and the remaining cost (2,970,183,600 Kyats and 2,966,225,542 Kyats, respectively) was paid by the government. Figure 1 represents the cost structures of two hospitals. The largest component of the total hospital cost for PMN GH was equipment, while medicines and medical supplies were the largest cost component for MTH, which was ranked as the second largest at PMN GH. The cost for human resource was the second largest component at MTH, but the fourth at PMN GH.

Table 4 shows the distributions of capital and recurrent costs for both hospitals. The cost difference of medical equipment and human resource were found between the two hospitals. The cost for equipment was higher and cost for human resource was lower at PMN GH than MTH. Other cost components were nearly the same amount between the two hospitals. As shown in Table 5, the eye unit consumed the largest amount (17.61%), while dental unit consumed the smallest amount (1.01%) of the total cost at PMN GH. On the other hand, the surgery unit was the largest consuming unit (25.11%) and dental unit was the smallest consuming unit (2.88%) at MTH. The costs were similar between the other four major units of each hospital—medicine, pediatrics, OG, and orthopedics.

**Table 4:** Composition of different cost components at Pyinmanar General Hospital (PMN GH) and Magway Teaching Hospital (MTH) in 2015–2016

Cost component	PMN GH			MTH		
	Cost		%	Cost		%
	Kyats	USD		Kyats	USD	
Capital						
Building	584,122,156	568,212.2	(19.35)	515,863,946	501,813.2	(17.32)
Medical equipment	857,302,473	833,951.8	(28.39)	611,489,144	594,833.8	(20.53)
Office equipment	696,935	677.9	(0.02)	772,649	751.6	(0.03)
Vehicle	22,440,160	21,828.9	(0.74)	24,155,561	23,497.6	(0.81)
Furniture	14,655,916	14,256.7	(0.49)	8,318,108	8091.5	(0.28)
Total capital cost	1,479,217,640	1,438,927.7	(48.99)	1,160,599,408	1,128,987.8	(38.97)
Recurrent						
Human resource	566,706,148	551,270.6	(18.77)	815,844,591	793,623.1	(27.39)
Medicines and medical supplies	805,862,066	783,912.5	(26.69)	857,289,953	833,939.6	(28.78)
Maintenance	82,189,945	799,51.3	(2.72)	80,817,840	78,616.6	(2.71)
Fuel	3,599,793	3501.7	(0.12)	3,919,600	3812.8	(0.13)
Electricity	20,593,550	20,032.6	(0.68)	40,563,900	39,495.0	(1.36)
Utilities	4,081,470	3970.3	(0.14)	8,816,500	387,953.8	(0.30)
Other	57,186,031	55,628.4	(1.89)	10,491,890	10,206.1	(0.35)
Total recurrent cost	1,540,219,003	1,498,267.5	(51.01)	1,817,744,274	1,670,957.5	(61.03)
Total cost of the hospital	3,019,436,643	2,937,195.2	(100)	2,978,343,682	2,897,221.5	(100)

1 USD = 1028 Kyats (on 2nd April, 2015)

**Table 5:** Cost of different cost centres at Pyinmanar General Hospital (PMN GH) and Magway Teaching Hospital (MTH) in 2015–2016

Cost center	PMN GH			MTH		
	Cost		%	Cost		%
	Kyats	USD		Kyats	USD	
Medicine	392,900,381	382,198.8	(13.01)	339,085,870	329,850.1	(11.39)
Surgery	364,393,431	354,468.3	(12.07)	747,825,919	727,457.1	(25.11)
Obstetrics and gynecology	424,387,912	412,828.7	(14.06)	367,802,061	357,784.1	(12.35)
Pediatrics	381,742,738	371,345.1	(12.64)	170,998,791	166,341.2	(5.74)
Orthopedics	417,418,671	406,049.3	(13.82)	418,067,149	406,608.1	(14.04)
Eye	531,752,888	517,269.3	(17.61)	165,086,175	160,589.7	(5.54)
ENT^a^	158,906,290	154,578.1	(5.26)			
Oncology				150,122,008	146,033.1	(5.04)
Dental	30,595,755	29,762.4	(1.01)	85,887,309	83,547.9	(2.88)
OPD^b^	317,338,577	308,695.1	(10.51)	533,468,400	518,938.1	(17.91)
Total	3,019,436,643	2,937,195.2	(100)	2,978,343,682	2,897,221.5	(100)

1 USD = 1028 Kyats (on 2nd April, 2015)

^a^Ear, nose and throat

^b^Outpatient department

**Fig. 2:**
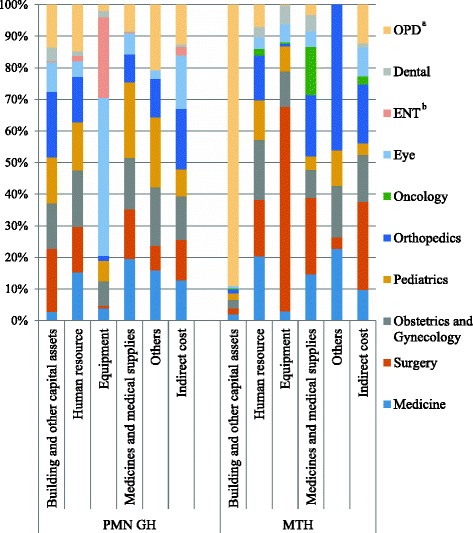
Contribution of the cost components by each cost center at Pyinmanar General Hospital (PMN GH) and Magway Teaching Hospital (MTH) in 2015–2016. The following five components constituted: 1) buildings and other capital assets, 2) human resource, 3) equipment, 4) medicines and medical supplies, and 5) others. The contributions of each cost component by the intermediate and the final cost centers were presented in this study. At PMN GH, the eye unit was the main contributor to equipment cost, while it was the surgery unit at MTH. In patient units and the operation theatre almost equally contributed to human resource. At MTH, OPD accounted for higher building cost

Figure 2 shows the contribution of costs of each cost component by cost centers at the hospitals. In MTH, the OPD represented the largest component of the cost for building and other capital assets, while the surgery unit represented the largest component of the cost for equipment, and the surgery and orthopedics unit for medicines and medical supplies. In PMN GH, the largest components of the cost for equipment were the eye unit, while the pediatric and medicine unit for medicines and medical supplies. The cost for the human resource was almost equally shared by five major inpatient units and the operation theatre at both hospitals. The contributions of direct and indirect costs in each final cost center for both hospitals are shown in Table 6. The OPD unit had the largest cost for buildings and other assets, while medicines and medical supplies represented the largest cost for the oncology unit.

**Table 6:** Contribution of direct and indirect cost in final cost centers of Magway Teaching Hospital (MTH) and Pyinmanar General Hospital (PMN GH) in 2015–2016

Final cost centers	Total cost	Building and other capital assets	Human resource	Equipment	Medicines and medical supplies	Others	Indirect cost
	Cost (Kyats)	(%)	(%)	(%)	(%)	(%)	(%)
MTH							
Medicine	338,809,626	(2.27)	(35.71)	(2.44)	(31.08)	(0.26)	(28.23)
Surgery	747,216,685	(1.02)	(14.26)	(24.82)	(23.36)	(0.02)	(36.53)
OG^a^	367,502,423	(2.84)	(31.01)	(8.79)	(17.18)	(0.17)	(40.01)
Pediatrics	170,859,483	(4.57)	(43.66)	(13.12)	(18.27)	(0.25)	(20.12)
Orthopedics	417,726,561	(1.21)	(20.33)	(0.54)	(33.38)	(0.42)	(44.11)
Oncology	149,999,708	(1.09)	(8.22)	(0.86)	(73.11)		(16.72)
Eye	164,951,683	(1.28)	(13.04)	(9.90)	(21.52)		(54.27)
Dental	88,243,715	(0.96)	(22.37)	(19.69)	(42.44)		(14.54)
OPD^b^	533,033,798	(65.38)	(7.82)	(0.11)	(4.29)		(22.41)
PMN GH							
Medicine	392,900,381	(0.58)	(14.85)	(4.21)	(32.36)	(0.66)	(47.34)
Surgery	364,393,431	(4.53)	(15.16)	(0.95)	(27.88)	(0.35)	(51.13)
OG^a^	424,387,912	(2.80)	(16.15)	(7.87)	(25.05)	(0.71)	(47.44)
Pediatrics	381,742,738	(3.17)	(15.39)	(7.36)	(40.78)	(0.94)	(32.36)
Orthopedics	417,418,671	(4.09)	(13.17)	(1.67)	(13.88)	(0.47)	(66.72)
Eye	531,752,888	(1.46)	(3.55)	(40.57)	(8.03)	(0.08)	(46.31)
ENT^c^	158,906,290	(0.14)	(3.95)	(68.74)	(1.65)		(25.52)
Dental	30,595,755	(11.86)	(19.12)	(29.66)	(4.73)		(34.63)
OPD^b^	317,338,577	(3.52)	(17.80)	(2.66)	(17.31)	(1.06)	(57.64)

^a^Obstetrics and gynecology

^b^Outpatient department

^c^Ear, nose and throat

## Discussion

This is the first study to report the unit costs of healthcare services at 200-bed general hospitals in Myanmar from the provider perspective. The unit costs were found to vary depending on the utilization of hospital services, the available resources used by each cost center, and type of medical service. The unit costs of the specialty units were highest due to expensive equipment and fewer patients. The highest unit cost was found for the ENT unit at PMN GH and the eye unit at MTH among all cost centers. The surgery unit of MTH also had a higher unit cost compared to other general inpatient units due to the higher cost of medicines and medical supplies for surgical patients. It also handled urology patients. Interestingly, compared to PMN GH, the orthopedic unit of MTH had a higher unit cost, with higher cost for medicines and medical supplies and lower case load.

The findings of the cost structures showed that medicines and medical supplies was the largest component of the total cost of MTH, while it was the second largest component for PMN GH. This may be due to increased budget for medicines and medical supplies by the government starting from the financial year 2012–2013, in line with the provision for free-of-charge essential medicines, emergency cases, and maternal and pediatric cases to reduce out-of-pocket payment. For the financial year 2012–2013, the budget for medicines was increased 20-fold compared to the previous year [24]. This is in line with studies from Pakistan and Vietnam, which also reported that medicines and medical supplies represented the second largest component [3, 25].

As higher cost of medicines and medical supplies may be responsible for higher unit cost, it is important to investigate the extent to which these medicines are used effectively and efficiently. The difference between the unit costs of orthopedic unit of the two hospitals also showed that hospital costs were also influenced by the choice of drugs by physicians. The quality of care and efficiency of the healthcare services also depends on rational drug use in hospitals. Therefore, standard treatment guidelines, monitoring of rational drug use, and promoting role of pharmacists are necessary for efficient use of the large budget for medicines and medical supplies.

The cost for equipment was the largest component at PMN GH, which was mainly contributed by procurement of new equipment for the eye unit during the period under study. However, the cost for equipment was the third largest component at MTH, which was mainly consumed by the operation theatre and surgery unit. A study of unit cost of different hospitals in India showed that a higher cost of equipment at tertiary care hospitals and relatively lower cost of equipment at lower level district hospitals [6]. This, it was found, was due to the advanced health services provided at tertiary care hospitals. As MTH is a tertiary level hospital, it may improve the utilization and performance of the hospital with increased investment in essential equipment. The higher maintenance and depreciation costs could become an avoidable burden if capital budgeting is flawed [26]. Higher maintenance cost should also be considered together with the higher capital budgeting. The results of this study also revealed that the maintenance cost was higher at PMN GH than MTH.

The hospital unit cost studies from developing countries reported that human resource was the largest component of the total cost of a hospital [5, 20, 25, 27, 28]. However, in this study, the cost for human resource was the second largest component at MTH, while it was the fourth largest at PMN GH. As MTH was a national level teaching hospital, more human resource—such as specialists and other staff, including teaching staff—were assigned, compared to PMN GH. This finding is in line with the hospital costing study in Malawi, which reported that salaries and wages below the true market level resulted in lower cost of manpower [29]. This finding was probably due to less manpower, lower salary, and smaller fringe benefits to the staff. Another study showed that higher investments in human resource, such as higher manpower or salaries, were associated with better performance, though it did not associate it with higher healthcare cost [30]. Thus, more investments should be directed toward human resource to improve hospital performance.

Among the final cost centers, the eye and ENT units consumed the largest cost at PMN GH, and its direct cost was mainly contributed by the cost for equipment. The inpatient day and number of admission to the eye unit was the second smallest unit, while the ENT ranked the smallest. Therefore, the unit costs of these two units were the highest. The eye unit performed a notable number of operation and outpatient cases during the year under study, while the ENT unit had very low output. The poor output of the ENT unit may be due to the fact that PMN GH is located near a 1000-bed tertiary level Nay Pyi Taw General Hospital, which provides more advanced and complete services for ENT patients. These findings indicated that it is important to consider capital budget allocation based on demand and output. As unit costs of PMN GH, eye and ENT units were extremely high, their utilization should be improved by increasing their services and specialized health professionals or reallocation of these costs to higher utilized hospitals. In MTH, the surgery unit consumed the largest cost because the cost of equipment and the cost of medicines and medical supplies used by the surgery unit were very high.

Regarding the cost of intermediate cost centers, MTH had three times higher numbers of laboratory tests and lesser numbers of patients taking X-ray tests than PMN GH, although both hospitals had the similar laboratory type. Therefore, the unit cost per laboratory test was much lower at MTH than at PMN GH. As the best usage of inputs at lower cost indicates efficiency, it showed that the laboratory unit of MTH was more efficient than the one at PMN GH [26]. Thus, the laboratory unit of MTH should be further strengthened, since the specialty services like urology and oncology are rarely available in other hospitals. During the period under study, the radiologist was not presented at MTH and the ultrasound scan could not be performed. The cost for radiology unit was only contributed by diagnosis by X-ray machine, and therefore, the unit cost per X-ray patient was obviously higher at MTH compared to PMN GH. Hence, a radiologist should be assigned for efficient utilization of cost and patient convenience. Both hospitals did not have well-functioning ICUs during the period under study, although they had ICU beds and some equipment for ICU. The ICUs of both hospitals should be strengthened to the standard level by identifying needs in order to avoid ineffective use of cost.

The total operating cost of PMN GH was higher than MTH due to the higher cost of medical equipment. Although MTH is a national level teaching hospital, the two hospitals had the same number of sanctioned beds and the hospital utilization rate was not significantly different. In India, the total operating cost of the tertiary hospital under study was higher than the district hospital [6]. The findings revealed that the output of the two hospitals did not have a remarkable difference in basic healthcare services. The main differences were found in specialty care, laboratory tests, and imaging tests. Although the number of admission cases was higher at PMN GH, compared to MTH, inpatient days were higher at MTH. The total recurrent cost was higher at MTH compared to PMN GH as the number of inpatient days is a good indicator for budget allocation. However, the capital investment was lower at MTH. Thus, government policy on capital investment should be reviewed and revised for efficient allocation.

The findings showed that the total cost of both hospitals was mainly contributed by the government, while donation accounted for 1.63% at PMN GH and 0.41% at MTH. Most of the donations were minor equipment, furniture, minor maintenance, and utility cost of each inpatient and outpatient unit. The donated amount was likely underestimated because some units did not practice proper record-keeping of donated items, and they were also concerned with misunderstanding regarding misuse of donations.

The study’s findings are similar to hospital costing studies from India and South Africa. These studies reported that the variation of unit cost depended on the volume of services [5, 6]. However, the unit costs of healthcare services at the two hospitals under study were higher compared to the unit cost at the 400-bed district hospital and 778-bed central hospital in India and the 170-bed district hospital and 980-bed central hospital in Vietnam [6, 25]. This may be because of the difference in the year of study, economic situations, allocation of resources at these hospitals, and utilization pattern of healthcare services in those countries.

The unit costs per inpatient day of medicine and pediatric unit were much lower at MTH because these units had higher inpatient day and lower total operating cost. Although these units used more cost for medicines and medical supplies, the equipment cost, which is a highly consumed hospital cost, was low for these units compared to other units. Furthermore, their utilization was similar to other units. The unit cost per OPD case was lower at PMN GH, with higher accessed cases. The OPD visit was higher at PMN GH because most cases were minor and simple cases. However, as MTH is a tertiary level hospital, most cases were complicated, including specialty OPD cases. The majority of the patients came from different regions where advanced care is unavailable, and therefore, the OPD visits were lower than at PMN GH. The higher costs of OPD at MTH were due to the establishment of a new building that was used during the study, and therefore, we did not consider the depreciation of building.

The unit cost per dental case was lower, with lower utilization rate, compared to MTH because it had lower total operating cost. This indicated that the unit cost was reduced by increasing utilization of each unit, and the unit cost was expensive when the total operating cost was high with lower utilization of services. The unit cost per PM&R case was relatively higher at PMN GH because of fewer patients. At the PM&R unit of PMN GH, only physiotherapists were available, with no physiatrists. However, it had high cost due to expensive physical therapy equipment. For effective utilization of this high cost, a physiatrist should be assigned or partly assigned from a nearby tertiary level hospital.

The study has some limitations. First, both hospitals had limited records on the utilization of laboratory and radiology tests by each unit. Therefore, the allocation of these cost centers to final cost centers was calculated by estimation of percentage distribution of total patients by experienced senior staff. Similarly, the maintenance and utility costs used for each unit were also poorly recorded. Hence, these costs were allocated to administrative cost centers. Thus, hospitals should maintain an exhaustive information management system for more reliable and detailed information, which would allow accurate assessment of the efficient utilization of resources [31]. Second, some variable costs for each department were underestimated because most of the inpatient departments contributed to select medical costs of financially underprivileged patients by cost sharing through other financially stable patients. The hospitals in this study had limited records for such costs.

## Conclusion

The unit costs were influenced by the utilization of hospital services by patients, efficiency of available resources, type of medical services, and physicians’ current medical practice. We found that the cost structures showed a variation pattern between two study hospitals. This was due to the difference in specialist services that were provided and level of hospital, even though they had a similar number of hospital beds. This study provided basic cost information to help policymakers in efficient allocation of healthcare budget within hospitals and the estimating budget for the establishment of a 200-bed hospital [32]. Moreover, the findings may be applied to estimate the cost of an essential package of health services, which was the goal of the Myanmar National Health Plan (2017–2021). The study findings are also useful for setting user fees packages for new health financing systems that seek to reduce OOP expenditure.

## Additional files

Additional file 1: Table S1. Allocation rules of assigning cost of each line item to cost centers. (DOCX 12 kb)

Additional file 2: Table S2. Allocation criteria for allocating the cost to final cost centers. (DOCX 12 kb)

## Abbreviations

ENT: Ear, nose and throat
ICU: Intensive care unit
MTH: Magway Teaching Hospital
OG: Obstetrics and gynaecology
OOP: Out-of-pocket expenditure
OPD: Outpatient department
PMN GH: Pyinmanar General Hospital
UHC: Universal health coverage
USD: The United States dollar
