# Supplementary material for: Unit cost of healthcare services at 200-bed public hospitals in Myanmar: what plays an important role of hospital budgeting?
Source: BMC Health Serv Res. 2017 Sep 19;17:669. doi: 10.1186/s12913-017-2619-z (PMC5605979; doi:10.1186/s12913-017-2619-z)
Supplement: Supplementary file 1 — Allocation rules of assigning cost of each line item to cost centers. (DOCX 12 kb) [file 12913_2017_2619_MOESM1_ESM.docx]

**Additional file 1: Table S1** Allocation rules of assigning cost of each line item to cost centers

| **Line item** | **Allocation rules** |
| --- | --- |
| Salary and allowances | Estimated spent of time in each cost centre according to duty roster |
| Building | Floor area of each cost centre |
| Furniture | Estimated actual use of each cost centre and assigned to administrative cost centre if there was no record |
| Vehicle | Allocated to administrative cost centre |
| Equipment | Estimated actual use of each cost centre |
| Medicines and medical supplies | Estimated actual use of each cost centre |
| Maintenance | Allocated to administrative cost centre |
| Materials and utilities | |
| Transport and labour | Allocated to administrative cost centre |
| Stationery | Allocated to administrative cost centre |
| Fuel | Allocated to administrative cost centre |
| Telephone bill | Allocated to administrative cost centre |
| Printing | Allocated to administrative cost centre |
| Water and electricity | Allocated to administrative cost centre |
| Taxes | Allocated to administrative cost centre |
| Utilities | Estimated actual use of each cost centre |
