# Supplementary material for: Unit cost of healthcare services at 200-bed public hospitals in Myanmar: what plays an important role of hospital budgeting?
Source: BMC Health Serv Res. 2017 Sep 19;17:669. doi: 10.1186/s12913-017-2619-z (PMC5605979; doi:10.1186/s12913-017-2619-z)
Supplement: Supplementary file 2 — Allocation criteria for allocating the cost to final cost centers. (DOCX 12 kb) [file 12913_2017_2619_MOESM2_ESM.docx]

**Additional file 2: Table S2** Allocation criteria for allocating the cost to final cost centers

| **Cost centres** | **Allocation criteria** |
| --- | --- |
| Administrative | Percentage distribution of total cost by other cost centres |
| Pharmacy | Estimated actual use of medicines and medical supplies by each cost centres |
| Laboratory | Percentage estimated by experienced staff |
| Radiology | Percentage estimated by experienced staff |
| Intensive care unit | Allocated to operation theatre |
| Operation theatre | Percentage distribution of total operations by final cost centres |
| Physical medicine and rehabilitation | Percentage distribution of total patients by final cost centres |
| Outpatient | Percentage distribution of total patients for other costs and distribution of human resource to general outpatient department |
